# Development and validation of Tagalog versions of the Drug Abuse Screening Test-20 (DAST-20) and Stimulant Relapse Risk Scale (SRRS) for drug users in the Philippines

**DOI:** 10.1371/journal.pone.0280047

**Published:** 2023-01-06

**Authors:** Takayuki Harada, Kazutaka Nomura, Toshiaki Baba, Tomohiro Shirasaka, Ayumi Takano, Shogo Kanamori

**Affiliations:** 1 Faculty of Human Sciences, University of Tsukuba, Tokyo, Japan; 2 School of Allied Health Sciences, Kitasato University, Kanagawa, Japan; 3 Bureau of International Health Cooperation, National Center for Global Health and Medicine, Tokyo, Japan; 4 Department of Psychiatry, Teine Keijinkai Medical Center, Hokkaido, Japan; 5 Department of Mental Health and Psychiatric Nursing, Tokyo Medical and Dental University, Tokyo, Japan; 6 Department of Community and Global Health, Graduate School of Medicine, The University of Tokyo, Tokyo, Japan; Drexel University, UNITED STATES

## Abstract

Methamphetamine use is becoming a major social issue in the Philippines, and this has been attracting international interest. Understanding the characteristics of drug users and the severity of their drug use is an urgent requirement for promoting effective treatment and support; however, in the Philippines, a lack of screening and assessment tools with confirmed reliability and validity is a major obstacle in this regard. Therefore, the aim of this study is to develop Tagalog versions of the Drug Abuse Screening Test-20 (DAST-20), a drug-abuse screening tool used worldwide, and the Stimulant Relapse Risk Scale (SRRS), a tool for quantitatively evaluating relapse among stimulant users, and to confirm their validity and reliability. Participants were 305 patients admitted to the Treatment and Rehabilitation Center (TRC) operated by the Philippines Department of Health for treatment for methamphetamine use. Sufficient internal consistency for the DAST-20 was confirmed, with a Cronbach’s alpha value of 0.81. Concerning validity, receiver-operating-characteristic analysis, featuring diagnoses from independent doctors, returned an acceptable area-under-curve value of 0.62. Sufficient internal consistency was also confirmed for the SRRS, with a Cronbach’s alpha value of 0.89. Correlation analysis of subjective drug craving (measured using a visual analog scale) and the SRRS revealed a significant positive correlation (*r* = 0.19, *p* < 0.001), confirming a certain level of validity. The Tagalog versions of the DAST-20 and SRRS developed in this study were confirmed to be reliable and valid. These scales could be effective for use in clinical settings and for research purposes.

## Introduction

The Philippines is one of the most seriously affected countries in the world by methamphetamine problems [1]. Domestic statistics estimate that there are approximately 1.8 million methamphetamine users in the country [2]. Along with the associated threats to the mental and physical health of drug users, such widespread drug use can result in serious socio-economic consequences, including fragmentation of families and local communities, increased crime, and obstruction of societal development [3].

Many countermeasures for drug use have been developed in the world, but the effects and costs vary. When the harms of drug use are converted into a monetary value and included in the overall social cost, it becomes apparent that the most expensive option is to do nothing; this is because a lack of action leaves the aforementioned harms unaddressed. According to an estimate by the Institute of Medicine [4], in the US, the drug-related cost to society is over $40,000 per person per year. Criminal sentencing and incarceration were once considered to be effective strategies for addressing drug use, but the effects of such policies have since been disproven [5,6]. In fact, “tough” methods of addressing drug problems, such as criminal punishment and detention, sever the social connections drug users have with their families and communities, and are likely to strengthen drug users’ criminal ties; further, this approach has a similar direct cost of almost $40,000 per year [4].

Currently, the most effective and low-cost strategy to address drug dependence, at just $2,700 per person, is to provide human services, including treatment, welfare, and education, within communities [4,7,8].

Soon after taking office as President of the Philippines in 2016, Rodrigo Duterte proclaimed a “war on drugs,” and launched a nationwide anti-drug campaign, setting this as the highest priority for his administration. In particular, President Duterte’s willingness to allow extrajudicial killing of drug users attracted criticism from around the world [9]; however, in addition to this extreme policy, he discussed providing community support, including treatment, for those who “turn themselves in.” As a result, over one million drug users surrendered to the authorities during the first six months of Duterte’s presidency, revealing the seriousness of the country’s drug problem [10].

When seeking to provide human services for drug users, it is first necessary to screen them to determine their demographic characteristics and the severity of their drug use. In the Philippines, not only is it impossible to provide the same treatment service to one million individuals, but it is also unrealistic to assume that they all require treatment; the level of dependence and seriousness of the problem can vary widely among persons. Therefore, there is an urgent need to screen these drug users using relatively simple and efficient methods, and to develop reliable tools for matching them with appropriate services.

The second step necessary in addressing this situation is providing appropriate services to individuals with drug problems. To this end, it is important, for clinical purposes, to understand the quality and validity of available services by quantitatively measuring their effectiveness. Further, because comparing the quality of a service between countries is a growing interest among researchers, it is essential to develop assessment tools that can be used worldwide without requiring alteration of their content (e.g., the number of items).

However, in the Philippines, screening and assessment tools with confirmed reliability and validity are limited. This is a critical issue in assessing the severity of the problem and matching treatment approaches accordingly. Moreover, this gap represents a major obstacle to conducting research in this field. The Drug Abuse Screening Test-20 (DAST-20) is one of the most widely used drug-abuse screening tools worldwide [11]. It is a self-administered questionnaire comprising 20 items and is capable of conveniently and quickly (within minutes) measuring the severity of drug use. This test can be used regardless of drug type, length of use, or frequency of use; for these reasons, the DAST-20 is widely used in clinical screening and research [12]. Thus, developing a Tagalog version of the DAST-20 and utilizing it in clinical settings and research in the Philippines can be expected to contribute to identifying effective solutions to the country’s drug problem. This would facilitate international comparison and help secure international support.

In evaluating the approaches of appropriate treatment services, it is important to assess not only the event of post-treatment relapse but also its risk. The Stimulant Relapse Risk Scale (SRRS) is a psychometric tool for estimating such a risk of relapse [13]. The SRRS is a self-administered scale developed in Japan to evaluate the level of craving for stimulants, which are the most problematic drugs in both Japan and the Philippines (particularly methamphetamines). As the scale focuses on the psychological mechanism of craving, it is useful for evaluating treatment programs that target craving. Further, the SRRS is clinically advantageous as it only includes a small number of items, allowing individuals with drug problems to easily evaluate their own level of craving and relapse risk [13]. Therefore, developing a Tagalog version of the SRRS is a pressing task for determining the validity of treatment services in the Philippines.

Accordingly, the aim of this study was to develop Tagalog versions of the DAST-20 and SRRS, two screening tools that are widely used internationally. As the key features of the DAST-20 are screening and evaluating severity, we aimed to verify the reliability and validity of these two objectives when developing the Tagalog version.

## Materials and methods

### Participants

As of April 2020, there were 14 Department of Health Drug Abuse Treatment and Rehabilitation Centers (TRCs) in the Philippines [14]. The participants of the present study were new patients admitted to the TRC located in the Manila metropolitan area for treatment for methamphetamine use; “new patients” were operationally defined as any patients admitted within the 30 days preceding the commencement of the survey or during the data-collection period. In principle, patients are admitted to the TRC on a voluntary basis or by a court order, and receive residential treatment [15].

The exclusion criteria for our sample were (1) being aged under 18 years, (2) being illiterate, (3) having abnormal mental and/or physical conditions that prevented appropriate participation in the study, and (4) being unwilling to participate.

According to the COSMIN Study Design Checklist for Patient-reported Outcome Measurement Instruments, a sample size of 200 or more is optimal for a cross-cultural validity study. Considering the number of new patients prior to the commencement of our study, the interim target was set at 150 participants, with the optimal goal of reaching 200 or more if possible. As a result, the final sample size was set at 300 patients.

The survey was administered between July 23 and September 23, 2019 either individually or in a group, after the completion of admission procedures.

### Patient and public involvement

Before the questionnaires were finalized, 10 patients volunteered to answer a draft version and offer feedback about any issues faced by them, including comprehension and the language of the questionnaire items. Through these iterative processes, the patients made high-value contributions to the development of the questionnaires and implementation of the survey.

### Development of the Tagalog version of the DAST-20 and SRRS

The DAST-20 is a 20-item self-administered test for evaluating severity in clinical settings and for treatment and assessment research. The test is designed to evaluate the severity of drug-abuse-related problems easily and practically. Respondents answer “yes” or “no” to questions, based on their experiences over the past 12 months; the test takes approximately five minutes to complete. Each item is scored either “1” or “0” points (two items are reverse scored), giving a total score between 0 and 20 points. Skinner and Goldberg reported that the English version of the DAST-20 has high internal consistency, with a Cronbach’s alpha coefficient of 0.74 [11]. The same study reported a five-factor structure, comprising “dependence,” “social problems,” “medical problems,” “polydrug abuse,” and “previous treatment.”

The SRRS is a self-administered scale developed to evaluate the level of drug craving—one of the primary symptoms of drug dependence—with the aim of treating such craving. The SRRS comprises five subscales (“anxiety and intention to use drug,” “emotionality problems,” “compulsivity for drug,” “positive expectancies and lack of control over drug,” and “lack of negative expectancy for the drug”), as well as auxiliary items, which are used to determine the extent of the patient’s awareness of their illness. This results in a total of 35 items. Respondents are asked to provide answers, using a five-point scale (“strongly disagree,” “disagree,” “neither agree nor disagree,” “strongly agree,” and “agree”), based on their experiences in the past week. Both “strongly disagree” and “disagree” are assigned one point, respectively, “neither agree nor disagree” is assigned two points, and both “strongly agree” and “agree” are assigned three points, respectively. Concerning the internal consistency of the SRRS, Ogai et al. [13] found the Cronbach’s alpha coefficients for each subscale to be 0.55–0.82.

The following procedure was adopted when developing the Tagalog versions of the above scales. First, after obtaining consent from the authors of the original scales, we contracted professional translators not part of the study team to perform translation from English to Tagalog. This translation was checked by an expert in drug addiction whose native language was Tagalog, and any unnatural sections or inappropriate terminologies were modified.

Next, the scales were back-translated into English by a different group of translation professionals. The original and back-translated versions were then checked for discrepancies by two researchers who specialized in drug addiction (non-Tagalog speakers), and by one research collaborator whose native language was Tagalog. Based on these noted discrepancies, the Tagalog version was revised before finalization. Additionally, the researchers and local professionals discussed the relevance and comprehensiveness of items. The finalized version was administered in a preliminary trial to 10 consenting TRC patients, who were asked whether any of the items were difficult to understand, and whether they encountered any difficulties when completing the questions. As a result, we found no further modification was necessary for the translated version.

### Measurements for concurrent validity

To verify the concurrent validity of the DAST-20, patients who were administered the DAST-20 were also independently diagnosed immediately after admission by doctors (who were not involved in the development or implementation of the Tagalog version of the DAST-20). The 13 doctors employed at the study facility each interviewed and examined participants individually. To ensure consistency, prior to the examination, all doctors were provided training regarding the Mini-International Neuropsychiatric Interview (MINI) method for substance-use disorders [16].

In their examinations, the doctors diagnosed, based on the MINI, whether the patients satisfied the Diagnostic and Statistical Manual of Mental Disorders (DSM-5) [17] criteria for substance-use disorder. The results of the DAST-20 administered at admission were concealed from the doctors at this time.

As an additional method for examining concurrent validity, after completing the DAST-20, patients rated their subjective drug craving using a visual analog scale (VAS). Specifically, participants were asked to rate, using a 10-centimeter horizontal line, their current level of drug craving; “none at all” was placed on the extreme left of the line, and “the highest possible craving” was placed on the extreme right.

### Questionnaire on drug experience and demographic factors

Participant demographics and related information were collected when administering the DAST-20. Items surveyed were sex, age, and primary drug used in their lifetime.

### Statistical analysis

Item-total (IT) correlation analysis and Cronbach’s alpha coefficients were calculated to determine the reliability of the Tagalog versions of the DAST-20 and SRRS. Then, confirmatory factor analysis was conducted to examine the construct validity of each scale. For the DAST-20, receiver operating curve (ROC) analysis, in conjunction with the doctors’ diagnoses, was conducted to determine criterion-related validity for screening. Finally, correlation coefficients with the VAS scores were calculated to determine, for each scale, the concurrent validity for craving severity.

### Ethical considerations

The study was approved by the University of Santo Tomas Graduate School Ethics Review Committee (GS-2017-PN Ex-01-R2) in the Philippines, and the University of Tsukuba Faculty of Human Sciences Ethical Committee (T29-15) in Japan.

Prior to the survey, all potential study participants were verbally informed the details of the study, including objectives, procedures, and handling of personal information, and were also provided the written explanation of this information. Written informed consent was obtained from all participants for inclusion in the study. Participants were further assured that they could withdraw their consent at any time during the study without negatively affecting their treatment. The questionnaire form was entirely anonymous, and no personal information was collected.

## Results

### Participants’ basic statistics

Descriptive statistics are shown in Table 1. Of the 336 participants, 31 met our exclusion criteria. Consequently, data for the remaining 305 participants were analyzed. There were 228 male and 77 female participants, with an overall mean age of 35.62 ± 9.90 years. For 182 participants, the primary drug used over the past 12 months was methamphetamine, while for eight it was marijuana.

**Table 1:** Participants’ characteristics.

Items	Values
Number of participants	305
Male	228
Female	77
Age (M ± SD)	35.62 ± 9.90
**Primary substance used over the past 12 months**	
Stimulant	182
Marijuana	8
Did not use any substance	115

### Reliability of the DAST-20

IT correlation analysis results are shown in Table 2. The total score for the Tagalog version of the DAST-20 had a significant positive correlation with all items (*r* = 0.20–0.64). Cronbach’s alpha coefficient was 0.81.

**Table 2:** Item means and item-total correlations for the Drug Abuse Screening Test-20.

Item	Item means	Standard deviations	Item-total correlations
1	0.49	0.50	0.35**
2	0.17	0.38	0.38**
3	0.17	0.38	0.42**
4	0.09	0.29	0.32**
5	0.10	0.30	0.20**
6	0.30	0.46	0.51**
7	0.62	0.49	0.50**
8	0.80	0.40	0.42**
9	0.73	0.44	0.55**
10	0.52	0.50	0.48**
11	0.57	0.50	0.62**
12	0.48	0.50	0.64**
13	0.47	0.50	0.62**
14	0.31	0.46	0.56**
15	0.29	0.45	0.57**
16	0.58	0.49	0.30**
17	0.25	0.44	0.45**
18	0.20	0.40	0.44**
19	0.38	0.49	0.44**
20	0.28	0.45	0.34**

**p < 0.01.

### Factor analysis for the DAST-20

The confirmatory factor analysis results for the DAST-20 are shown in Table 3. Confirmatory factor analysis was conducted for a model of the correlations between factors, and the chi-square goodness of fit test returned significant results (chi-square = 400.84, *df* = 305, *p* < 0.001). Standardized path coefficients from the subscales to each item were 0.21–0.44 for “dependence,” 0.039–0.74 for “social problems,” 0.41–0.54 for “medical problems,” 0.22–0.57 for “polydrug abuse,” and 0.29–0.56 for “previous treatment.” Model fit indices were as follows: goodness of fit (GFI) = 0.882, adjusted GFI (AGFI) = 0.854, comparative fit index (CFI) = 0.771, root mean square residual (RMR) = 0.012, and root mean square error of approximation (RMSEA) = 0.070.

**Table 3:** Confirmatory analysis and model fit of the Drug Abuse Screening Test-20.

	**Standardized path coefficients**				
**Item no.**	**Subscale 1: Dependence**	**Subscale 2: Social problems**	**Subscale 3: Medical problems**	**Subscale 4: Polydrug abuse**	**Subscale 5: Previous treatment**
17	0.44				
4	0.43				
5	0.21				
12		0.74			
13		0.73			
11		0.62			
9		0.52			
15		0.49			
10		0.45			
8		0.39			
6			0.54		
18			0.41		
14				0.57	
3				0.47	
2				0.37	
1				0.25	
16				0.22	
7					0.56
19					0.51
20					0.29
**Correlation between subscales**					
	**Subscale 1**	**Subscale 2**	**Subscale 3**	**Subscale 4**	**Subscale 5**
Subscale 1		0.56	0.93	0.91	0.56
Subscale 2			0.69	0.72	0.66
Subscale 3				0.98	0.73
Subscale 4					0.63

*N* = 305, χ^2^ = 400.84, *df* = 160, *p* < 0.001, goodness of fit index = 0.882, adjusted goodness of fit index = 0.845, comparative fit index = 0.771, root mean square residual = 0.012, root mean square error of approximation = 0.070.

### ROC analysis of the DAST-20

**Fig 1:**
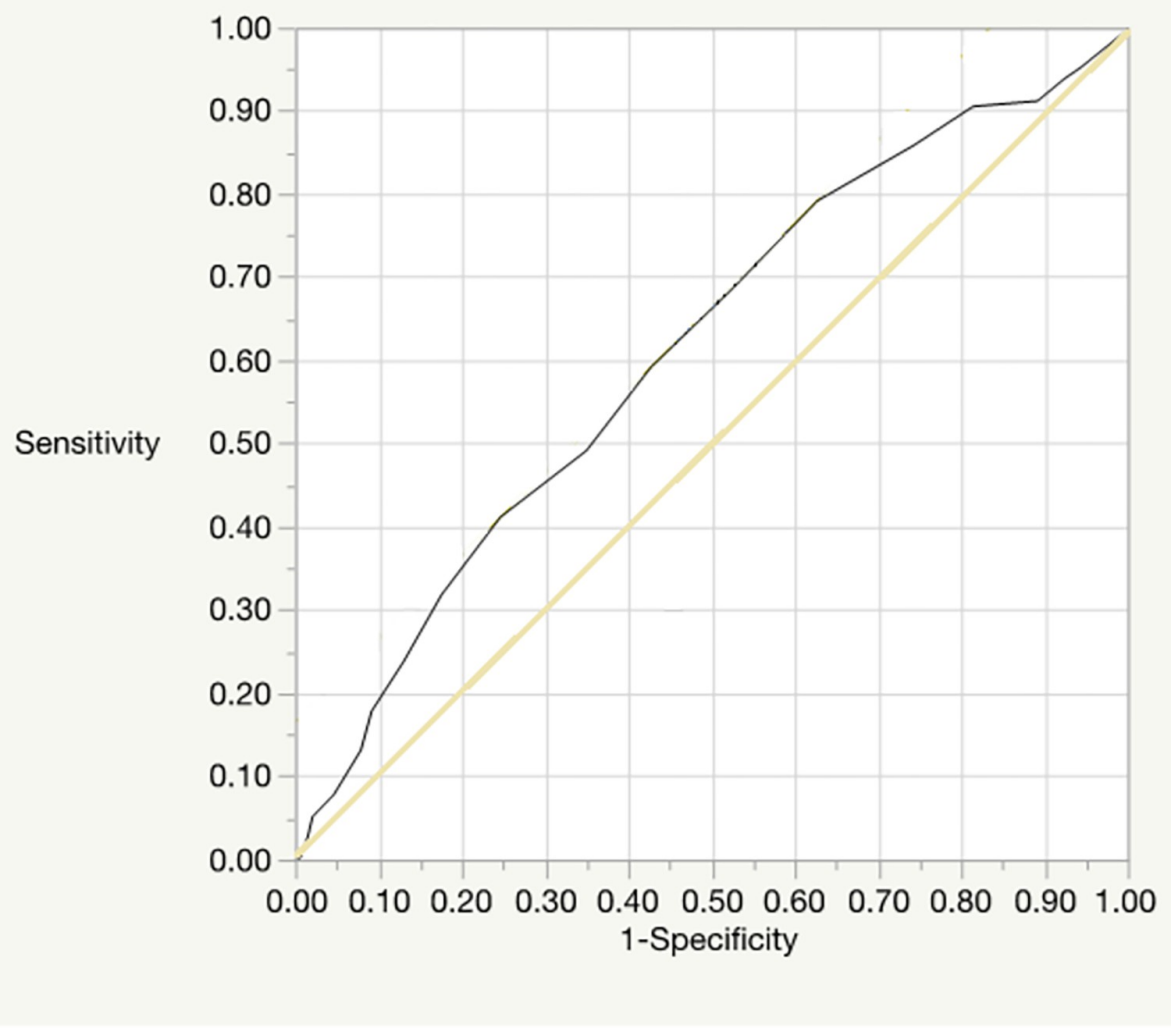
The ROC curve for drug dependence.

**Fig 2:**
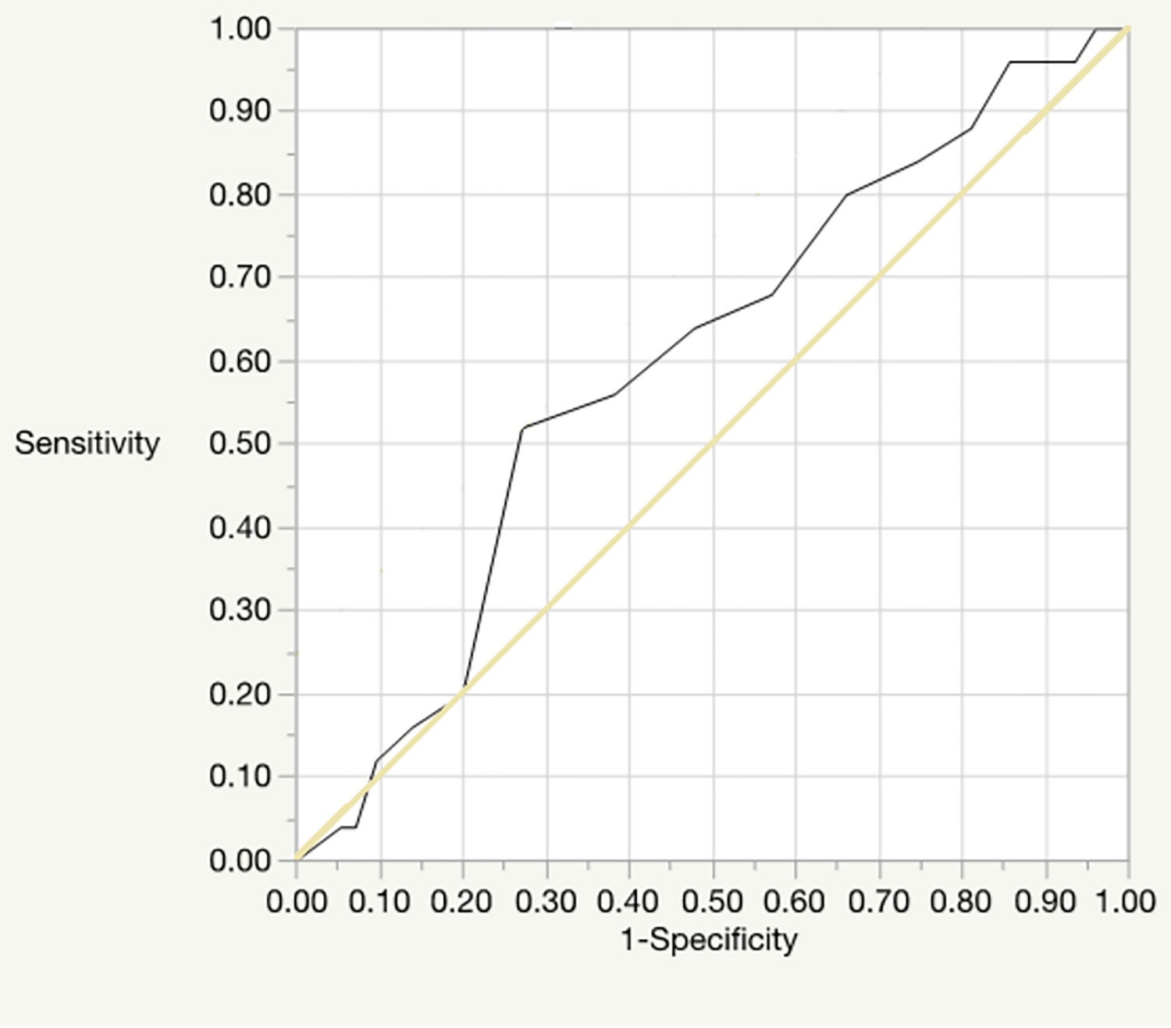
The ROC curve for drug abuse.

ROC analysis was conducted for both the DAST-20 total score and the doctors’ diagnoses of patients regarding drug dependence and drug abuse; the doctors’ diagnoses were performed based on the diagnostic criteria for substance-use disorder provided in the DSM-5 [18] and the MINI. Figs 1 and 2 show the respective ROC curves for drug dependence and drug abuse. The area-under-curve (AUC), indicating the sensitivity of the drug dependence ROC curve was 0.62 (95% confidence interval [CI] = 0.55–0.68, *p* < 0.001), and an optimal cutoff value of 10 was recommended. The AUC of the drug abuse ROC curve was 0.40 (95% CI = 0.29–0.51, *n*.*s*.), and an optimal cutoff value of 5 was recommended.

### Concurrent validity of the Tagalog version of the DAST-20

Correlation analysis with the subjective drug-craving VAS revealed a significant positive correlation (*r* = 0.31, *p* < 0.001).

### Reliability of the SRRS

IT correlation analysis results are shown in Table 4. Excluding items 9, 14, and 21, for which there was no significant correlation, and item 17 (*r* = −0.32), for which a negative correlation was found, the total score of the Tagalog version of the SRRS had a significant positive correlation with all other items (*r* = 0.15–0.79). Excluding the auxiliary items concerning awareness of one’s illness, the positive correlations were 0.21–0.79. Taking all items into consideration, Cronbach’s alpha coefficient was 0.89; excluding auxiliary items, Cronbach’s alpha coefficient was 0.90.

**Table 4:** Item means and item-total correlations of the SRRS.

Item	Item means	Standard deviations	Item-total correlations
1	1.55	0.76	0.61**
2	1.82	0.85	0.49**
3	1.54	0.77	0.45**
4	1.53	0.84	0.15**
5	1.80	0.83	0.36**
6	2.02	0.86	0.28**
7	1.62	0.77	0.52**
8	1.43	0.73	0.60**
9	1.73	0.83	0.07
10	1.98	0.82	0.37**
11	1.44	0.79	0.26**
12	1.43	0.75	0.21**
13	1.36	0.69	0.21**
14	1.84	0.89	0.03
15	1.33	0.69	0.31**
16	2.04	0.86	0.35**
17	2.31	0.82	−0.32**
18	1.43	0.68	0.73**
19	1.75	0.84	0.51**
20	1.48	0.73	0.68**
21	1.57	0.82	0.08
22	1.26	0.61	0.67**
23	1.56	0.75	0.60**
24	1.38	0.67	0.72**
25	1.69	0.78	0.55**
26	1.66	0.79	0.56**
27	1.42	0.70	0.74**
28	1.31	0.62	0.79**
29	1.49	0.75	0.68**
30	1.46	0.72	0.68**
31	1.27	0.63	0.72**
32	1.55	0.77	0.66**
33	1.33	0.66	0.74**
34	1.32	0.67	0.71**
35	1.32	0.65	0.65**

**p < 0.01.

### Factor analysis of the SRRS

Confirmatory factor analysis was conducted for a model concerning the correlations between factors (Table 5), and the chi-square goodness of fit test was significant (χ^2^ = 861.86, *df* = 305, *p* < 0.001). Standardized path coefficients from the subscales to each item were −0.02 to 0.82 for anxiety and intention to use drug, 0.45 to 0.76 for emotionality problems, 0.65 to 0.82 for compulsivity for drug use, 0.69 to 0.77 for positive expectancies and lack of control over drug, and 0.27 to 0.68 for lack of negative expectancy for drug use. Model fit indices were GFI = 0.839, AGFI = 0.811, CFI = 0.893, RMR = 0.033, and RMSEA = 0.062.

**Table 5:** Confirmatory analysis and model fit for the SRRS.

	**Standardized path coefficients**				
**Item no.**	**Subscale 1:** **Anxiety and intention to use drug**	**Subscale 2:** **Emotionality problems**	**Subscale 3:** **Compulsivity for drug use**	**Subscale 4:** **Positive expectancies and lack of control over drug**	**Subscale 5:** **Lack of negative expectancy for drug use**
33	0.82				
27	0.76				
35	0.74				
22	0.74				
1	0.66				
2	0.51				
6	0.35				
12	−0.02				
23		0.76			
25		0.67			
19		0.60			
7		0.59			
3		0.57			
5		0.48			
10		0.44			
16		0.45			
28			0.82		
34			.78		
31			.76		
8			0.65		
24				0.77	
18				0.76	
30				0.75	
29				0.74	
20				0.69	
32				0.69	
17					0.68
14					0.44
9					0.27
21					0.27
**Correlation between subscales**					
	**Subscale 1**	**Subscale 2**	**Subscale 3**	**Subscale 4**	**Subscale 5**
Subscale 1		0.87	1.00	0.99	−0.61
Subscale 2			0.86	0.82	−0.75
Subscale 3				1.00	−0.63
Subscale 4					−0.58

*N* = 305, χ^2^ = 861.86, *df* = 395, *p* < 0.001, goodness of fit index = 0.839, adjusted goodness of fit index = 0.811, comparative fit index = 0.893, root mean square residual = 0.033, root mean square error of approximation = 0.062.

### Concurrent validity of the Tagalog version of the SRRS

Correlation analysis with the subjective drug-craving VAS revealed a significant positive correlation with the SRRS total score (*r* = 0.19, *p* < 0.001). Results for each subscale were as follows: “anxiety and intention to use drug,” *r* = 0.25 (*p* < 0.001); “emotionality problems,” *r* = 0.16 (*p* < 0.01); “compulsivity for drug use,” *r* = 0.16 (*p* < 0.01); “positive expectancies and lack of control over drug,” *r* = 0.16 (*p* < 0.01); and “lack of negative expectancy for drug use,” *r* = −0.07 (*n*.*s*.).

## Discussion

The aim of this study was to develop Tagalog versions of the DAST-20 and SRRS.

To develop the DAST-20, we conducted an IT correlation analysis and calculated Cronbach’s alpha coefficient to estimate the scale’s reliability. As a result, we found significant positive correlations between the total scores and scores for each item. This result indicates that the item and scale scores were consistent; participants who scored highly on the overall scale scored similarly highly on each item. Cronbach’s alpha coefficient was 0.81, which is not particularly high when compared to a previous study of the DAST-20 that reported a reliability of 0.74–0.95; however, because the value exceeds 0.80, the Tagalog version can be considered to have sufficient reliability [19].

Further, confirmatory factor analysis conducted to examine the scale’s construct validity yielded GFI and AGFI scores in excess of 0.800, confirming the overall goodness of fit [20]. Although CFI did not reach 0.800, its value of 0.771 can be considered to indicate that the data and the model’s goodness of fit were within an acceptable range. Further, both RMR and RMSEA were close to 0 and thus were considered to be within an acceptable range. From the above results, we determined the Tagalog version of the DAST-20 to have the same factor structure as that of the original as well as a sufficient level of construct validity.

To consider the DAST-20’s concurrent validity regarding screening, we also conducted ROC analysis with doctors’ diagnoses (based on the MINI). The AUC difference for substance-dependence diagnosis was significant; although the value in question (0.62) indicated low accuracy, it nevertheless confirmed that the tool can contribute to screening for substance dependence. An optimal cutoff score of 10 was recommended, which is higher than the 3–5 range reported in Cocco and Carey [19]. Additionally, the results were not statistically significant for diagnosis of drug abuse, meaning this tool cannot be used for screening cases of drug abuse. Further, the results of the correlation analysis, performed to test concurrent validity regarding drug-abuse severity, found a significant positive correlation with subjective drug craving. Although the value in this regard (0.31) was not high, it showed a correlation with subjective craving and supports the scale’s concurrent validity.

To develop the Tagalog version of the SRRS, the key features of which are evaluating relapse risk and craving, we also conducted IT correlation analysis and calculated Cronbach’s alpha coefficient to estimate reliability. Consequently, we found significant positive correlations between the total score and scores for each item, excluding those from the subscale “lack of negative expectancy for drug use.” This result indicates that participants who score highly on the overall scale will similarly score highly on each item, excluding items concerning the subscale “lack of negative expectancy for drug use.”

Although the inconsistency between the items for “lack of negative expectancy for drug use” and the total score remains an issue, validity testing performed during the development of the original version of the SRRS also found no significant correlation between “lack of negative expectancy for drug use” and the other factors [13]. Thus, our results match those obtained when testing the validity of the original scale.

Cronbach’s alpha coefficient for all items was 0.89, increasing to 0.90 when auxiliary items were excluded. Based on past research regarding the reliability of the SRRS, which found a Cronbach’s alpha value of 0.86 for all items [13], the Tagalog version can be considered to have sufficient reliability.

Confirmatory factor analysis to verify the construct validity of the scale found that GFI and AGFI exceeded 0.800, confirming the overall goodness of fit [20]. Further, CFI was 0.893, also exceeding 0.800; thus, the data and model can be considered to have a good fit. Both RMR and RMSEA were close to 0 and thus were considered to be within an acceptable range. From the above results, the Tagalog version of the SRRS was found to have the same factor structure as that of the original and a sufficient level of construct validity. However, several items did not reach the 0.40 standard that is considered to indicate acceptable factor loading [21]. Nonetheless, the aim of this study was to develop a scale usable for international comparison, and the Tagalog version of the SRRS uses the same items as the original version; thus, this issue may persist for other versions. Exploration of items with low factor loadings is a task for future studies.

Correlation analysis to test concurrent validity for craving found a positive correlation with total score and all subscale scores, aside from “lack of negative expectancy for drug use.” This confirms that the higher the SRRS score, the higher the drug craving. Concerning “lack of negative expectancy for drug use,” a similar lack of correlation with subjective drug craving was found in previous research [13]; thus, our result confirms that the Tagalog scale has the same concurrent validity as the original.

Based on the above results, we believe we have succeeded in developing tools applicable for evaluating the validity of services and screening for substance dependence in the Philippines.

Despite the fact that there has been a large number of drug users in the Philippines for many years, prior to this study there were no associated psychometric tools suitable for clinical and research purposes. In this study, we developed tools capable of screening drug users’ treatment needs and substance dependence, and objectively measuring relapse risk and treatment effects. These tools are significant in five important ways. First, having a simple and self-administered method of screening for severity of drug use will enable health professionals to quickly consider and provide services appropriate for patients’ conditions. Second, it will be possible to prevent wastage of medical and social resources and to optimize the costs of services. Third, health professionals can quantitatively measure results during and after receiving services, allowing them to determine the validity of such services. Fourth, researchers worldwide can simultaneously compare the scope of the problem and its solution at an international level, and also share the current status and direction of future solutions. Fifth, providing effective assessment and service will incentivize scientific and humanitarian solutions for drug users and reduce the application of unsuitable treatments.

As the data for this research were collected from the patients of a single TRC, the generalizability of these findings to all TRC patients in the Philippines and all individuals with drug dependence may be limited. Although the primary targets of the Philippines’ support system for drug abuse are patients at TRCs, if these tools are to be used with those with substance-use disorder living in the community, their reliability and validity should be examined using a community-sourced sample.

## Conclusion

Due to the dearth of drug-dependence research resources in the Philippines, the Tagalog versions of the DAST-20 and SRRS were developed to be used as assessment tools to form a foundation of drug-dependence research. Furthermore, this research confirmed the reliability and validity of both of these tools and found that they can serve as effective tools for use in clinical and research contexts. In the future, it is expected that further evidence for their reliability and validity will be accumulated through comparison with tools that measure the severity of other mental illnesses and the findings of predictive studies involving relapse data.

## Supporting information

S1 Checklist (DOCX)

S1 File Tables Confirmatory analysis and model fit of the DAST-20 and the SRRS. (DOCX)

S2 File (DOCX)
