# Supplementary material for: Development and validation of Tagalog versions of the Drug Abuse Screening Test-20 (DAST-20) and Stimulant Relapse Risk Scale (SRRS) for drug users in the Philippines
Source: PLoS One. 2023 Jan 6;18(1):e0280047. doi: 10.1371/journal.pone.0280047 (PMC9821478; doi:10.1371/journal.pone.0280047)
Supplement: S1 File — (DOCX) [file pone.0280047.s002.docx]

**Tables**

Table 1. Confirmatory analysis and model fit of the DAST-20

|  | Standardized path coefficients | | | | |
| --- | --- | --- | --- | --- | --- |
| Item no. | Subscale 1 Dependence | Subscale 2 Social problems | Subscale 3 Medical problems | Subscale 4 Polydrug abuse | Subscale 5 Previous treatment |
| 17 | 0.44 |  |  |  |  |
| 4 | 0.43 |  |  |  |  |
| 5 | 0.21 |  |  |  |  |
| 12 |  | 0.74 |  |  |  |
| 13 |  | 0.73 |  |  |  |
| 11 |  | 0.62 |  |  |  |
| 9 |  | 0.52 |  |  |  |
| 15 |  | 0.49 |  |  |  |
| 10 |  | 0.45 |  |  |  |
| 8 |  | 0.39 |  |  |  |
| 6 |  |  | 0.54 |  |  |
| 18 |  |  | 0.41 |  |  |
| 14 |  |  |  | 0.57 |  |
| 3 |  |  |  | 0.47 |  |
| 2 |  |  |  | 0.37 |  |
| 1 |  |  |  | 0.25 |  |
| 16 |  |  |  | 0.22 |  |
| 7 |  |  |  |  | 0.56 |
| 19 |  |  |  |  | 0.51 |
| 20 |  |  |  |  | 0.29 |
| Correlation between subscales | | | | | |
|  | Subscale 1 | Subscale 2 | Subscale 3 | Subscale 4 | Subscale 5 |
| Subscale 1 |  | 0.56 | 0.93 | 0.91 | 0.56 |
| Subscale 2 |  |  | 0.69 | 0.72 | 0.66 |
| Subscale 3 |  |  |  | 0.98 | 0.73 |
| Subscale 4 |  |  |  |  | 0.63 |

Note:

*N* = 305, χ^2^ = 400.84, *df* =160, p < 0.001, GFI = 0.882, AGFI = 0.845, CFI = 0.771, RMR = 0.012, RMSEA = 0.070

GFI: goodness of fit index, AGFI: adjusted goodness of fit index, CFI: comparative fit index, RMR: root mean square residual, RMSEA: root mean square error of approximation

Table 2. Confirmatory analysis and model fit for the SRRS

|  | Standardized path coefficients | | | | |
| --- | --- | --- | --- | --- | --- |
| Item no. | Subscale 1:  Anxiety and intention to use drug | Subscale 2:  Emotionality problems | Subscale 3:  Compulsivity for drug use | Subscale 4:  Positive expectancies/lack of control over drug | Subscale 5:  Lack of negative expectancy for drug use |
| 33 | 0.82 |  |  |  |  |
| 27 | 0.76 |  |  |  |  |
| 35 | 0.74 |  |  |  |  |
| 22 | 0.74 |  |  |  |  |
| 1 | 0.66 |  |  |  |  |
| 2 | 0.51 |  |  |  |  |
| 6 | 0.35 |  |  |  |  |
| 12 | −0.02 |  |  |  |  |
| 23 |  | 0.76 |  |  |  |
| 25 |  | 0.67 |  |  |  |
| 19 |  | 0.60 |  |  |  |
| 7 |  | 0.59 |  |  |  |
| 3 |  | 0.57 |  |  |  |
| 5 |  | 0.48 |  |  |  |
| 10 |  | 0.44 |  |  |  |
| 16 |  | 0.45 |  |  |  |
| 28 |  |  | 0.82 |  |  |
| 34 |  |  | 0.78 |  |  |
| 31 |  |  | 0.76 |  |  |
| 8 |  |  | 0.65 |  |  |
| 24 |  |  |  | 0.77 |  |
| 18 |  |  |  | 0.76 |  |
| 30 |  |  |  | 0.75 |  |
| 29 |  |  |  | 0.74 |  |
| 20 |  |  |  | 0.69 |  |
| 32 |  |  |  | 0.69 |  |
| 17 |  |  |  |  | 0.68 |
| 14 |  |  |  |  | 0.44 |
| 9 |  |  |  |  | 0.27 |
| 21 |  |  |  |  | 0.27 |
| Correlation between subscales | | |  |  |  |
|  | Subscale 1 | Subscale 2 | Subscale 3 | Subscale 4 | Subscale 5 |
| Subscale 1 |  | 0.87 | 1.00 | 0.99 | −0.61 |
| Subscale 2 |  |  | 0.86 | 0.82 | −0.75 |
| Subscale 3 |  |  |  | 1.00 | −0.63 |
| Subscale 4 |  |  |  |  | −0.58 |

Note:

*N* = 305, χ^2^ = 861.86, *df* = 395, p < 0.001, GFI = 0.839, AGFI = 0.811, CFI = 0.893, RMR = 0.033, RMSEA = 0.062

GFI: goodness of fit index, AGFI: adjusted goodness of fit index, CFI: comparative fit index, RMR: root mean square residual, RMSEA: Root mean square error of approximation.
